# PPARs in Regulation of Paraoxonases: Control of Oxidative Stress and Inflammation Pathways

**DOI:** 10.1155/2012/616371

**Published:** 2012-01-24

**Authors:** Jordi Camps, Anabel García-Heredia, Anna Rull, Carlos Alonso-Villaverde, Gerard Aragonès, Raúl Beltrán-Debón, Esther Rodríguez-Gallego, Jorge Joven

**Affiliations:** Centre de Recerca Biomèdica, Hospital Universitari de Sant Joan, Institut d'Investigació Sanitària Pere Virgili, Universitat Rovira i Virgili, 43201 Reus, Spain

## Abstract

The paraoxonase (PON) group of enzymes, composed of PON1, PON2, and PON3, play an important role in decreasing oxidative stress by degrading lipid peroxides. PON1 synthesis is upregulated by PPAR. Several pharmacological compounds (acting as antioxidants and, hence, atheroprotective) stimulate both PPAR activity and PON1 expression. Recent evidence suggests that PON1 and the monocyte chemoattractant protein-1 (MCP-1) are involved in coordinating the inflammatory response in damaged tissues; PPAR may be central in the regulation of these biochemical pathways. This article reviews the state of knowledge on PON1 biochemistry and function, the influence of genetic variation, and the regulation of PON1 expression by pharmaceutical compounds that increase PPAR activity. We also describe recent lines of evidence suggesting links between PON1 and MCP-1 and how their production may be regulated by PPAR.

## 1. Introduction

Nuclear receptors are a large group of transcription factors which are important as master regulators of genes involved in metabolic control. Hence, an intensive search for ligands for these receptors is underway so as to design improved preventive and therapeutic strategies that target these ligands/receptors in an effort to combat diseases which are direct determinants of the human lifespan. Among these receptors are the peroxisome proliferator-activated receptors (PPARs). The name is confusing because they do not induce proliferation of peroxisomes in humans, but which has been described in rodents [1]. When this receptor was cloned, it was termed PPAR*α* [2]. PPAR*β*/*δ*  and *γ* were identified subsequently as structural homologues of PPAR*α*, which were shown to control the expression of other metabolic genes. The PPAR subfamily of nuclear receptors all bind as heterodimers with RXR to peroxisome proliferator response elements in the target gene, especially those involved in the homeostatic control of metabolism and in the defense against toxic compounds. Of particular importance is the association of these receptors with the risk of cancer, cardiovascular diseases, diabetes and dementia, as well as inflammation and oxidative stress responses that underlie these diseases. In this scenario, it is relevant to review the relationships between PPAR and the antioxidant enzymes collectively termed paraoxonases (PONs).

## 2. The Paraoxonases and Lipid Peroxide Degradation

The PON protein family comprises 3 enzymes, PON1, PON2, and PON3 genes coding which are located close to each other on chromosome 7q21-22 [3, 4]. PON1 and PON3 genes are expressed in most tissues, and their protein products are found in circulation bound to high-density lipoprotein (HDL) [5–7]. Conversely, PON2 is an intracellular enzyme which is not found in plasma [8]. PON1 was first identified by Aldridge in 1953 who, while examining the rates of hydrolysis of organophosphate insecticides in different tissues of rats and rabbits [9, 10], observed that rabbits had a very high rate of paraoxon degradation in serum, and that this compound was cleaved by an esterase. Aldridge segregated esterases into two categories according to whether they were inhibited by interaction with substrates (B-esterases) or whether they could hydrolyze substrates (A-esterases). Based on these original publications, the serum A-esterase was referred to as paraoxonase because of its ability to hydrolyze the toxic oxon metabolite of parathion, hence “paraoxon”. When two closely similar enzymes were identified in the mid-1990s, the original paraoxonase was referred to as to PON1, while the two new enzymes were termed PON2 and PON3 [3].

The ability to hydrolyze paraoxon was employed in the 1960s as the method to measure PON1 activity in several species and tissues. It soon became apparent that, in human serum, there was considerable variability between individuals with respect to PON1 activity. Frequency distribution histograms found three different phenotypes, corresponding to high, intermediate, and low serum PON1 activities. Also, the frequency for the low activity allele varied significantly between populations of different geographical or ethnic origin, a higher frequency being observed in Caucasian than in African, Asian, or Australian aborigine populations [11].

 Over subsequent years, several assays were developed to examine the PON1 phenotype polymorphism [12]. Plotting the values of hydrolysis of one substrate (whether phenyl acetate, diazoxon, or sarin) against that of a second substrate (paraoxon) provided a clear resolution of the individuals with low (termed AA), intermediate (termed AB), and high (termed BB) PON1 activities [13]. Eckerson et al. [14, 15] noted that the B isozyme was more active in the presence of NaCl. Using this property, the authors plotted arylesterase activity values against paraoxonase activity values measured in the presence of 1 M NaCl. This system was shown to be the most efficient in differentiating between different PON1 polymorphisms.

The genetic bases of PON1 phenotype polymorphisms were first defined by Adkins et al. [16] who sequenced the coding region for PON1 from human cDNA libraries and identified two polymorphic sites: Arg/Gln at position 192 (*PON1_192_* polymorphism, with two alleles termed Q and R), and Leu/Met at position 55 (*PON1_55_* polymorphism, with two alleles termed L and M). *PON1_192_* polymorphism correlated clearly with the AB phenotype system, QQ individuals segregated with the AA phenotype while RR individuals segregated with the BB phenotype.

**Figure 1:**
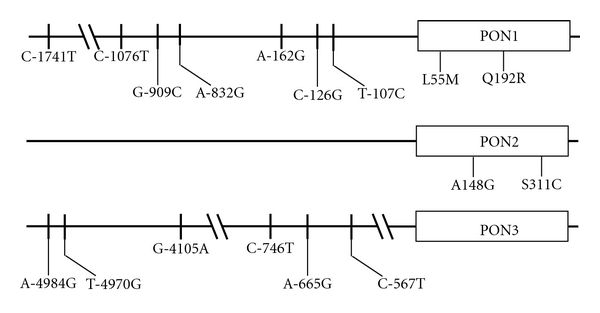
Schematic representation of the most prevalent polymorphisms of the PON1, PON2, and PON3 genes.

 In 1997, Blatter Garin et al. [17] evaluated the influence of *PON1_192_* and *PON1_55_* polymorphisms on the enzyme's activity as well as its concentration. They observed considerable differences in relation to *PON1_55_* genotype, individuals carrying a leucine at position 44 (L isoform) having higher serum PON1 concentrations than those with a methionine (M isoform) at this position. In contrast, the *PON1_192_* polymorphism affected enzymatic activity but had only a slight impact on the concentration of PON1 in serum. These findings suggested a possible divergence between the enzyme's concentration and that of its activity. More recently, several polymorphisms in the promoter region of the *PON1 *gene have been described (Figure 1). However, only *PON1_−108_*, *PON1_−909_,* and* PON1_−1741_* appear to be significantly associated with changes in the enzyme's concentration or activity in serum. 

**Figure 2:**
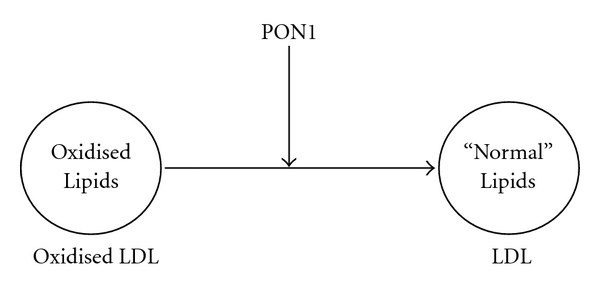
The antioxidant role of PON1. The main physiological role of this enzyme is to degrade oxidized phospholipids and oxidized cholesteryl esters in lipoproteins.

**Figure 3:**
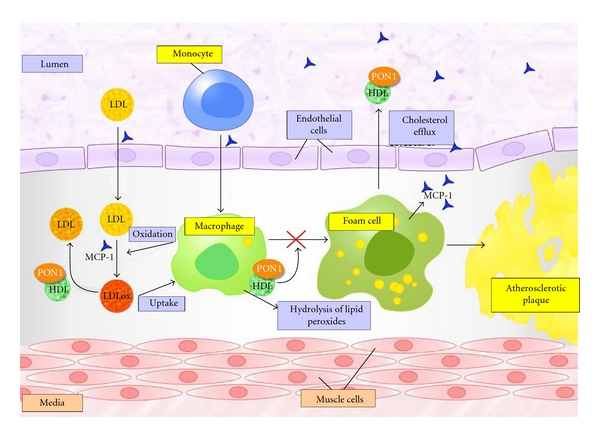
The protective role of PON1 in atherosclerosis. Circulating monocytes are activated in an oxidant and inflammatory environment and become macrophages. This environment also promotes the oxidation of LDL particles, which are internalized into the macrophages that become foam cells. PON1 hydrolyzes oxidized lipids in LDL, reversing this lipoprotein to its natural status and, thus, inhibiting the development of atherosclerosis. PON1, by inhibiting the production of MCP-1, is anti-inflammatory and favors cholesterol efflux from macrophages.

 The first approximation to the possible physiological role of PON1 came from experiments by Mackness et al. [18]. Since paraoxon and other toxic chemicals are clearly not present in the human body under normal circumstances, other molecules would need to be the physiological substrates of this enzyme. These authors investigated, using purified PON1, the protection against copper-induced oxidation of low-density lipoprotein (LDL) that is provided by HDL (Figure 2). They observed that HDL as well as PON1 prevented lipoperoxide generation during the process of LDL oxidation. This implied that the enzyme itself may be involved in the protective function attributed to HDL. Further studies provided evidence that PON1 protects LDL and HDL from lipid peroxidation by degrading specific oxidized cholesteryl esters and specific oxidized phospholipids contained in oxidized lipoproteins [19–25]. Experimental animal studies provided further support to the *in vitro* experiments, the data showing that the physiological function of PON1 is to hydrolyze oxidized lipids and, hence, function as an antioxidant enzyme. Decreased serum PON1 activities and increased oxidative stress were observed in apolipoprotein E-deficient mice as well as in dyslipidemic obese mice [26]. Perhaps the most conclusive data were generated in the PON1-deficient mouse model and the human-PON1 transgenic mouse model, PON1 plus apolipoprotein E double-deficient mice showing increased lipoprotein oxidation and atherosclerosis than the apolipoprotein E-alone deficient mice. HDL isolated from PON1-deficient mice was unable to prevent LDL oxidation in cultured arterial tissue, in contrast to HDL obtained from control mice. Avian HDL (the lipoprotein does not contain PON1) is unable to protect LDL from oxidation. In agreement with these observations, overexpression of human PON1 in transgenic mice inhibited lipid peroxide formation in HDL and protected LDL structure and function [27–30]. A scheme of the role played by PON1 in protecting against oxidative stress and atherosclerosis is shown in Figure 3.

PON1 is, in turn, inactivated by oxidized lipids. This was shown by Aviram et al. [31], who demonstrated that incubating PON1 *in vitro *with oxidized palmitoyl arachidonoyl phosphatidylcholine, lysophosphatidylcholine, and oxidized cholesteryl arachidonate resulted in inactivated PON1 arylesterase activity, as well as did oxidized LDL. Cysteine-284 was required for this effect of oxidized lipids on PON1 because, in recombinant PON1 in which mutation of this amino acid had been induced, no inactivation was observed. Further investigation by the same research group showed that, under oxidative stress, PON1 may be inactivated by *S*-glutathionylation, a redox regulatory mechanism characterized by the formation of a mixed disulfide between a protein thiol (i.e., cysteine-284) and oxidized glutathione [32].

## 3. PON1 Native Activity Is Lactonase

PON1 hydrolyzes a broad range of substrates including esters, lactones, organophosphates such as the nerve agents soman and sarin, lipid peroxides, and estrogen esters [12, 13, 33, 34]. In addition, PON1 metabolizes certain drugs and has been proposed for therapeutic use in drug inactivation [35, 36]. Identifying the native function of PON1 has been hampered, for a considerable time, by confusion with respect to the structure and mechanism-of-action of this enzyme. Purified PON1 preparations are unstable and often contaminated. However, the method of “directed evolution” has been productive in determining PON1 structure and function. Essentially, directed evolution seeks to replicate the evolutionary process in the laboratory by artificially inducing mutations in the gene-of-interest, followed by selection and amplification of the variants which show an enhancement of the desired characteristics. Using this technique, Harel et al. [37] described PON1 as a six-bladed beta propeller with a unique active site lid that is also involved in HDL binding. The active site and the deduced catalytic mechanism suggested that PON1 is reminiscent of the secreted phospholipase A2. Despite huge increase in complexity of living organisms during evolution, relatively little novelty has been produced at the molecular level since primordial times [38]. PONs appeared very early in evolution and are present in many organisms, from invertebrates to mammals [12]. Jensen [39] proposed that, in contrast to more evolutionary-modern enzymes, primitive enzymes possessed very broad specificities, and that it is this catalytic versatility that enabled a relatively few enzymes to perform the multitude of functions necessary to maintain the ancestral organism [40–43]. 

Hence, research on PON1 function was focused on trying to distinguish the native or “ancestral” function of this enzyme from all other secondary or “adapted” functions. Again, directed evolution studies, together with structure-function studies, established the primordial function of PON1 as that of a lipolactonase [44–47] which subsequently evolved new substrate specificities. These studies also established that the preferred substrates of PON1 are 5- and 6-membered-ring lactones, typically with aliphatic side-chains [38].

 New data on the PON1 mechanism of lactone hydrolysis have been reported by Tavori et al. [48] using modeling and docking simulation techniques. These methods use theoretical models of the ligands being evaluated (in this case, lactones) which are allowed to interact with models of the protein's three-dimensional structure. This enables the nature of the target ligand, as well as the fitness of the evaluated ligand within the protein, to be determined. The authors suggested that PON1 active site may be reached by a range of lactones that have similar orientation in the active centre. The carboxylate moiety is directed towards the hydrophylic inner part of the active centre, while the ligand's aromatic ring is facing the outer hydrophobic part. The results from Tavori et al. also revealed an inverse correlation between docking energy and rate of lactone hydrolysis, as well as a direct correlation with the length of lactone side chain.

**Figure 4:**
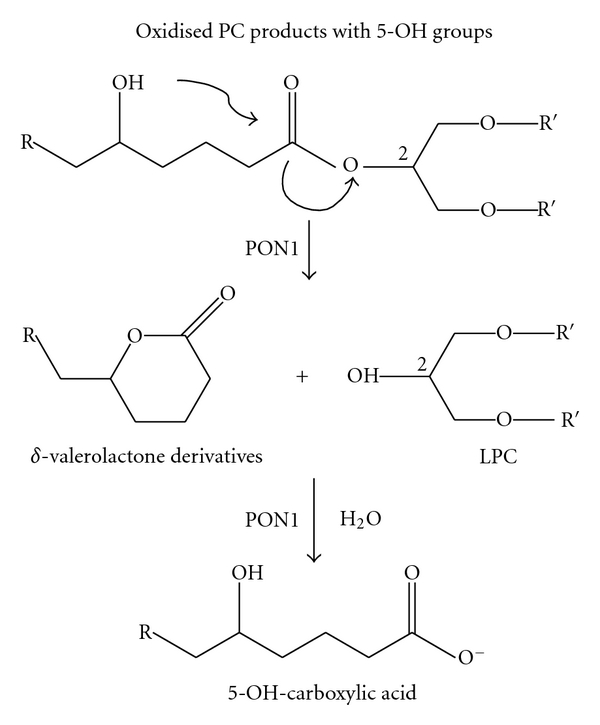
Proposed mechanism for the hydrolysis of oxidized lipids in macrophages by PON1 to yield lysophosphatidylcholine. Oxidized lipids with hydroxyl groups at the 5′ position could be lactonized by PON1 to yield lysophosphatidylcholine and the corresponding *δ*-valerolactone products. The latter can be hydrolyzed again by PON1 to yield the corresponding 5-hydroxycarboxylic acid.

 Recently, kinetic and site-directed mutagenesis studies demonstrated that the His^115^–His^134^ dyad is necessary for PON1 lactonase activity and, as well, for oxidized lipid degradation [49, 50]. A model has been proposed to link PON1 capability with that of lipid peroxide degradation [50]. According to this model, oxidized lipids containing hydroxyl groups at the 5′-position could be lactonized by PON1 to yield lysophosphatidylcholine and *δ*-valerolactone products (Figure 4). As such, according to this hypothesis, the PON1 ability to degrade lipid peroxides is secondary to its lipolactonase activity.

## 4. The Role of PON2 and PON3

Currently, not much is known about PON2 and PON3 proteins. Their genes were identified in 1996 when Primo-Parmo et al. [3] identified a large number of cDNA sequences in the Genome Data Base with significant homology to, but not identical with, human PON1. The percentage identity among human PON1, PON2, and PON3 genes is similar (about 70%) and the genes are believed to derive from a common precursor [51]. Ng et al. [8] demonstrated that PON2 is not present in the circulation, although its gene expression was detected in several human tissues. The authors concluded that this represents ubiquitous intracellular distribution of the enzyme. They also reported that cells transfected with the human PON2 gene have a higher antioxidant capacity than those cells that were not transfected. PON3 is present in HDL and prevents lipoproteins from oxidation *in vitro*. PON2 and PON3 also have genetic polymorphisms in their promoter and codifying regions that influence their protein expression (Figure 1). Both PON2 and PON3 are able to hydrolyze lactones, but not paraoxon or other xenobiotics [34, 52, 53], that is, all three PON enzymes have lactonase activity and hydrolyze lipid peroxides, but only PON1 has esterase activity (Table 1). 

## 5. PON1 and PPAR***α***

Fibrates are hypolipidemic drugs that act via activation of PPAR*α*. Their main therapeutic function is to decrease serum triglyceride concentrations. A mild increase in HDL-cholesterol concentration is also achieved. PPAR*α* activators induce the expression of apolipoprotein A1, the main apoprotein of HDL, and of the ATP-binding cassette of A1 (ABCA1), a transporter complex controlling cellular cholesterol efflux [54].

There have been conflicting reports on the influence of fibrate therapy on serum PON1 levels. Increase in enzyme activity appears to depend on type, and perhaps dosage, of fibrate employed. Durrington et al. [55] observed that bezafibrate and gemfibrozil, administered for 8 weeks, failed to influence serum PON1 activity in type IIb hyperlipidemic patients. Tsimihodimos et al. [56] found that 3-month treatment with micronized fenofibrate did not influence PON1 levels in types IIa, IIb and IV dyslipidemic patients. Conversely, Paragh et al. [57] observed that a 3-month administration of gemfibrozil increased serum PON1 activity in patients with hypertriglyceridemia. This research group found that ciprofibrate administration increased HDL-cholesterol concentration and serum PON1 activity in patients with metabolic syndrome [58]. In rats receiving a fructose-enriched diet (an experimental model of liver steatosis and metabolic syndrome), bezafibrate reduced oxidative stress and increased serum PON1 levels [59]. A recent report described that micronized fibrate increased the activity and concentration of PON1 and reduced oxidized LDL levels in dyslipidemic patients with low HDL-cholesterol levels. Interestingly, this effect was independent of *PON1* gene polymorphisms [60]. There are several potential PPAR*α* binding sites in the *PON1* gene promoter. However, Gouédard et al. [61] did not observe any increase in *PON1* gene expression following PPAR*α* activation, and this suggested that the mechanism of promoter activation induced by fibrates does not involve this nuclear receptor.

## 6. PON1, PON2, and PPAR***γ***

Rosiglitazone is a PPAR*γ* agonist that improves insulin sensitivity and glycemic control, stimulates reverse cholesterol transport, and reduces inflammation in individuals with type 2 diabetes [62–64]. In a randomized, cross-over, placebo-controlled, double-blind clinical trial, rosiglitazone was shown to increase fasting PON1 activity and to attenuate the postprandial fall in PON1 activity. However, the concentration of serum PON1 was observed not to change significantly [65]. A combination of rosiglitazone and metformin improved insulin resistance and fat distribution abnormalities (lipodystrophy) in patients infected with the human immunodeficiency virus (HIV) [66]. Our group reported that both treatments increased fasting and post-prandial serum PON1 activity and decreased plasma concentrations of the monocyte chemoattractant protein-1 (MCP-1) in HIV-infected patients undergoing highly active antiretroviral therapy [67]. Results from these studies indicated that plasma HDL-cholesterol concentrations did not significantly change. These data suggested that the observed effects on PON1 were independent of HDL synthesis. We also reported that metformin activates the peroxisome proliferators-activated response coactivator-1*α* and regulates oxidative stress homeostasis [68].

Several studies have shown that statins, widely used pharmacological compounds for the treatment of hyperlipidemia, activate PPAR*γ*, and that this activation is associated with increases in PON1 expression. Tomàs et al. [69] were the first to report that simvastatin administration (20 mg/day for 4 months) increased serum PON1 activity in hypercholesterolemic patients. The increases were modest (about 12% on average) and were accompanied by significant decreases in serum cholesterol and lipid peroxides, as well as LDL-cholesterol concentrations. They did not find any significant modulation associated with HDL-cholesterol levels or with *PON1_192_* and *PON1_55_ DNA *polymorphisms. Simvastatin attenuated myocardial inflammation in rats that had cardiopulmonary bypass surgery, and the phenomenon was associated with an increase in PPAR*γ* expression [70]. Atorvastatin and rosuvastatin have also been shown to increase PPAR*γ* [71] and PON1 expressions. Harangi et al. [72] observed that atorvastatin (10 mg/day for 6 months) increased serum PON1 activity in hypercholesterolemic patients, with changes in lipid profile and oxidative stress similar to those described by Tomàs et al. (described above). Kassai et al. [73] also confirmed that atorvastatin (20 mg/day for 3 months) increased serum PON1 activity. This statin has been shown to increase serum PON1 activities in experimental rabbits fed a high-cholesterol diet [74]. However, Bergheanu et al. [75] reported that atorvastatin (increasing doses up to 80 mg/day for 18 weeks) did not modify serum PON1 activity, although rosuvastatin administration (increasing doses up to 40 mg/day for the same period of time) was associated with a significant increase in serum PON1 activity. A recent detailed clinical report by Mirdamadi et al. [76] was that of a study conducted with 164 hypercholesterolemic patients subdivided into three groups to receive atorvastatin (10 mg/day, *n* = 61), simvastatin (10–20 mg/day, *n* = 46), or fluvastatin (80 mg/day, *n* = 57) for a period of 3 months. The results indicated that all three statins were able to increase serum PON1 activity, albeit moderately. 

 Of note is the study by Shiner et al. [77] which found that antioxidant polyphenols obtained from some plants increased PON1 and PON2 expression through PPAR*γ* activation. Pomegranate juice and its polyphenols (punicalagin and gallic acid) increased PON2 expression in cultured macrophages, and this phenomenon was associated with activation of PPAR*γ* and AP-1. Similarly, the incubation of hepatocytes with polyphenols from pomegranate juice increased PON1 expression via the PPAR*γ*-PKA-cAMP pathway [78]. 

## 7. PON1 and PPAR***δ***

**Figure 5:**
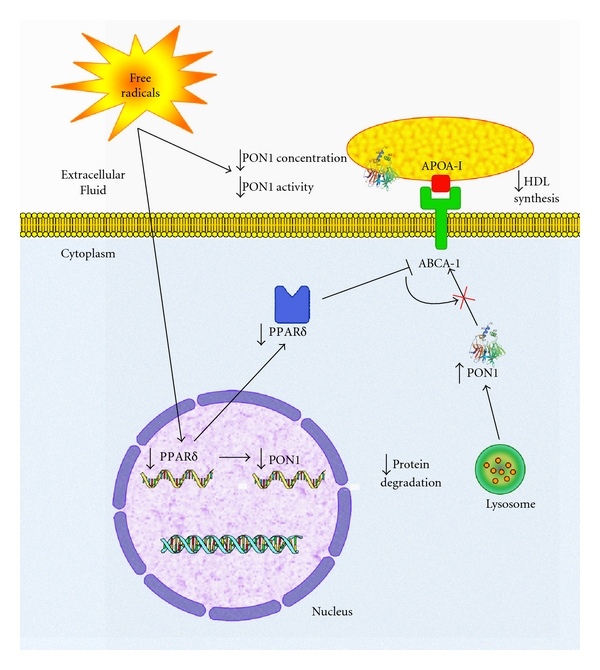
A hypothetical biochemical pathway that could explain the PON1 alterations observed in rats with experimental cirrhosis. Free-radical-induced liver impairment would result in a decrease in *PPAR*δ** gene expression and, as a consequence, in *PON1 *gene expression. It would also induce an inhibition of ABCA1, a decrease in HDL synthesis, and, therefore, a decrease in serum PON1 concentration. Intrahepatic PON1 levels would be increased as a consequence of decreased protein degradation. This figure is reproduced from [79].

There is a dearth of information on the possible relationships between PPAR*δ* and PON1. Our group reported [79] that rats with experimental liver cirrhosis had a significant decrease in hepatic PPAR*δ* and PON1 expression which was associated with inflammatory and fibrogenetic reactions. However, hepatic PON1 protein concentration was increased as a consequence of a decreased degradation of the enzyme (Figure 5). We did not observe any significant change in PPAR*α* and PPAR*γ* expression. These results suggested an involvement of PPAR*δ* in the regulation of oxidative stress in chronic liver impairment. To the best of our knowledge, this is the only published report showing an association between this transcription factor and PON1.

## 8. Combined Functioning of PON1 and MCP-1 in Regulating Inflammatory Response: A Role for PPAR?

The upregulation of MCP-1 production by oxidized lipids and lipoproteins is an important factor in the initial stages of inflammation [80, 81]. Mackness et al. [82] demonstrated that PON1, when incubated with endothelial cells, inhibited the production of MCP-1 induced by oxidized LDL. Indeed, several lines of evidence recently published by our group suggest that PON1 and MCP-1 act collaboratively in regulating inflammatory processes. PON1 and MCP-1 protein expressions are observed in conjunction in most normal and diseased tissues, while increased MCP-1 concentration and decreased PON1 activity are often observed in conditions involving oxidative stress [7, 83, 84].

Lysophosphatidylcholine (LPC) is a prominent component of oxidized LDL. During oxidation, 40% of LDL phosphatidylcholine can be converted to LPC by LDL-associated phospholipase A2. LPC stimulates the cellular production of MCP-1 at the transcription level through a mechanism that involves MEK/ERK, tyrosine kinase, and (to a lesser extent) protein kinase C (PKC) [85]. More recent data suggest that 12/15-lipoxygenase (12/15LO) is required for early onset high-fat-diet-induced adipose tissue inflammation and insulin resistance in mice [86]. Cells overexpressing 12/15LO secrete an excess of MCP-1 and, correspondingly, adipose tissues from 12/15LO knockout (KO) mice fed a high-fat diet are not infiltrated by macrophages, do not show any increase in inflammatory markers, and do not exhibit changes in insulin-stimulated glucose disposal rate or of hepatic glucose output.

 An interesting hypothesis is that PPARs are intimately involved in regulating and coordinating PON1 and MCP-1 expression. Considerable evidence (highlighted here in after) shows that PPARs upregulate PON1 expression in a variety of clinical and experimental situations. Recent evidence indicates that PPAR downregulates MCP-1 expression. Simvastatin decreases serum MCP-1 concentration in rats with cardiopulmonary bypass surgery and stimulates myocardial PPAR*γ* levels [70]. Similarly, rosuvastatin and atorvastatin increases PPAR expression and attenuates atherosclerosis mice deficient in apolipoprotein E [71]. Propofol, a sedative with antioxidant properties, decreases oxidative stress and MCP-1 production and increases PPAR*γ* in mice with sepsis-induced acute kidney injury [87]. Quercetin attenuates inflammation, decreases MCP-1 production, and improves insulin resistance in human-cultured adipocytes. This compound also counteracts tumor necrosis factor-induced inhibition of PPAR*γ* expression in these cells [88]. Rosiglitazone, which is a ligand for PPAR*γ*, inhibits inflammation and reduces MCP-1 production by murine cells [89] and has similar effects on lipopolysaccharide-treated mice and HK-2 cells [90]. Finally, telmisartan, an angiotensin type I receptor blocker, increases PPAR*γ* activity and PPAR ligand-binding activity, reduces atherosclerosis in mouse macrophages [91], and reduces MCP-1 production by peripheral monocytes in patients with essential hypertension [92].

## 9. Conclusion

It seems well established that PPARs, especially PPAR*γ*, are important factors in the regulation of PON1 expression, and in counteracting oxidative stress. Evidence exists that they also contribute to the control of inflammation by downregulating the expression of MCP-1. Several pharmaceutical preparations eliciting a beneficial antioxidant, anti-inflammatory, and atheroprotective function increase PON1 expression and decrease that of MCP-1. These data suggest that PPARs play a key role in the regulation of oxidation and inflammation and, as such, drugs that stimulate PPAR activity may be important tools in the struggle against diseases related to these biochemical alterations.

## Figures and Tables

**Table 1:** Principal properties of the PON enzymes.

Enzyme	Gene expression	Protein expression	Present in circulation	Lactonase activity	Hydrolysis of lipid peroxides	Esterase activity
PON1	Ubiquitous	Ubiquitous strong	HDL	Yes	Yes	Yes
PON2	Ubiquitous	Ubiquitous weak	No	Yes	Yes	No
PON3	Ubiquitous	Ubiquitous strong	HDL	Yes	Yes	No
